# Seasonal effects of the *UCP3* and the *RPTOR* gene polymorphisms on obesity traits in Japanese adults

**DOI:** 10.1186/1880-6805-33-38

**Published:** 2014-12-22

**Authors:** Kazuhiro Nakayama, Hiroshi Miyashita, Sadahiko Iwamoto

**Affiliations:** Division of Human Genetics, Center for Molecular Medicine, Jichi Medical University, Shimotsuke-shi, Tochigi, 329-0498 Japan; Jichi Medical University Health Care Center, Shimotsuke-shi, Tochigi, 329-0434 Japan

**Keywords:** Uncoupling protein 3 gene, Single nucleotide polymorphism, Visceral fat accumulation, Seasonality

## Abstract

**Background:**

Non-shivering thermogenesis (NST) involves a substantial amount of energy expenditure in humans and, thus, contributes to reducing the risk for obesity. Molecular evolutionary studies have reported that SNPs in/near the uncoupling protein 3 gene (*UCP3*) and the regulatory associated protein of mTOR complex 1 gene (*RPTOR*) might influence NST and confer adaptive advantages for modern human dispersal into cold environments. In the present study, the impact of these SNPs on obesity-related traits was investigated.

**Methods:**

Study subjects consisted of 2,834 Japanese adults (percentage of female: 46%, mean age: 51.5). Associations of the *UCP3*-55C/T and the *RPTOR*-26934C/T - the 2 potential genetic variations involved in cold adaptation and thermogenic mechanisms in mammals, with quantitative obesity-related traits including body mass index (BMI), waist circumference, visceral fat area (VFA), VFA adjusted for BMI, and selected blood parameters - were tested using multiple linear regression models. Sliding windowsampling analysis was applied to depict seasonal effects of the SNPs on the obesity-related phenotypes.

**Results:**

*UCP3*-55C/T and the *RPTOR*-26934C/T did not show any association with obesity traits and blood chemical parameters in multiple linear regression models consisting of the whole subjects. Moreover, sliding window sampling-based association analyses involving seasonality also failed to find associations between these two SNPs and obesity-related traits.

**Conclusions:**

*UCP3*-55C/T and the *RPTOR*-26934C/T may only have subtle effects on the development of obesity-related traits in the present humans. These two SNPs might be irrelevant to inter-individual variations in energy metabolism and efficiency of NST.

## Background

The principal cause of obesity in humans is the imbalance of energy intake and expenditure. Non-shivering thermogenesis (NST) currently draws much attention as a factor for reducing the risk of obesity through enhancement of energy expenditure. NST mainly occurs in thermogenic adipocytes including brown and beige/brite adipocytes (BAT) [1–4]. The amount of active BAT observed in the human body is strongly influenced by the age of the subject. The age-related decline of BAT is thought to increase risks for obesity in adults [5]. Additionally, activation of BAT has frequently been observed in experiments performed in cold seasons compared to hot seasons [2]. The seasonality of BAT activity would partly explain the enhancement of energy expenditure in winter [6, 7]. NST likely had an adaptive significance in the modern human dispersal into cold environments. The susceptibility to obesity in the present human populations is thus considered to have been partly shaped by past genetic adaptation to cold environments [8, 9].

A SNP near the uncoupling protein 1 gene (*UCP1*), namely *UCP1*-3826G/A, is a promising genetic variation linking cold adaptation and resistance to obesity. UCP1 is a mitochondrial protein highly expressed in BAT and dissipates the energy for ATP synthesis as heat. [10]. The A allele of the -3826 site, which increased the *UCP1* expression levels, was linked with higher BAT activity and reduced risks of obesity [10–13]. A worldwide survey of human genome variations revealed that the A allele was more prevalent in ethnic groups inhabiting higher latitudes. The cline was considered to be shaped by past adaptations to cold environments: the A allele was more adaptive at higher latitudes due to its enhanced activity in NST [14].

Besides the *UCP1*-3826G/A, genetic variations supporting the relationship between past cold adaptation and the present susceptibility to obesity have not been extensively studied [9]. Evolutionary genetic studies have reported several SNPs that showed striking correlation between allele frequencies and geographic or climatic factors [15–17]. Among them, SNPs near uncoupling protein 3 gene (*UCP3*-55C/T) and the regulatory associated protein of the mTOR complex 1 gene (*RPTOR*-26934C/T) were considered to be potential adaptive genetic variation candidates, since molecular and animal studies have suggested relevance of these 2 genes in thermogenic mechanisms in mammals. *UCP3* is a member of the uncoupling protein gene family and possibly takes part in the NST in skeletal muscles [18]. *RPTOR* encodes an intracellular signaling protein that participates in regulation of cell growth [19]. Adipocyte-specific knockout of *Rptor* (mouse homologue of *RPTOR*) induced enhanced mitochondrial respiration and lean phenotypes in mice [20]. These SNPs, therefore, may contribute to susceptibility to obesity similar to *UCP1*-3826G/A. In the present study, we performed genetic associations using a large-scale cohort to assess the impact of the *UCP3* and *RPTOR* SNPs on obesity-related traits in modern humans.

## Methods

### Subjects

The study population comprised 3,013 Japanese adults who attended general health checkups in the Jichi Medical University Healthcare Center during January 2009 to March 2011[8, 13]. All participants provided written informed consent. Medical information from the health checkup and genome DNA were obtained from all participants. Visceral fat area (VFA) was measured with the bioelectrical impedance method at the umbilical level [21]. Waist circumference (WC) at the umbilical level was measured using a tape measure. Blood medical traits including fasting plasma glucose (FPG), hemoglobin (Hb) A_1c_, plasma triglycerides (TG), total cholesterol (TC), high density lipoprotein cholesterol (HDL-c) were directly measured using standard methods. Low density lipoprotein cholesterol (LDL-c) levels were estimated using the Friedewald’s formula. Individuals who had undergone abdominal surgery were excluded from the analyses. The study design was approved by the Ethical Committee of Jichi Medical University.

### Genotyping

Genotypes of *UCP3*-55C/T (rs1800849) and *RPTOR*-26934C/T (rs11868112) from each participant were determined using TaqMan SNP Genotyping Assays on a ViiA 7 Real Time PCR System (Thermo Fisher Scientific, Waltham, MA, USA), and KAPA PROBE FAST qPCR kit (Kapa Biosystems, Boston, MA, USA).

### Association analysis

*χ*^2^ goodness-of-fit test was used to assess the Hardy-Weinberg equilibrium of the genotype distribution. In the association analyses, an additive model was assumed. Association of SNP genotypes and quantitative traits was assessed using multiple linear regression models [13]. For body mass index (BMI), WC, and VFA, the number of T alleles (0, 1, and 2), sex, age, speed of walking (based on the health checkup questionnaire question: 'Do you walk faster than other people of same sex and about same ages?') of each participant [13] were included as explanatory variables. Associations of SNPs and obesity-related blood parameters including FPG, HbA1c, TG, TC, HDL-c and LDL-c were also tested. In addition to genotypes, sex, age, and BMI were also included as explanatory variables for the blood parameters. Seasonality of SNP effects on VFA was evaluated using the sliding window sampling, which was previously used to show the seasonal effect of *UCP1*-3826G/A on VFA. Briefly, associations of the SNPs with VFA were tested in 12 subsets of the populations, each consisting of individuals sampled during overlapping continuous 3-month periods. Monthly outdoor temperature data measured at the nearest observatory were obtained from the Japan Meteorological Agency. The above-described statistical tests were performed using SPSS version 22 (IBM Corporation, Armonk, NY, USA).

## Results and Discussion

In total, 2,834 successfully genotyped individuals, who had not undergone abdominal surgery, were included for further analyses. The characteristics of the genotyped subjects are summarized in Table 1. The genotype distribution of *UCP3*-55C/T was as follow: CC = 1,356, CT = 1,205, and TT = 273. No deviation from the Hardy-Weinberg equilibrium was observed for the genotype distribution (*P* = 0.824). *RPTOR*-26934C/T genotypes were as follow: CC = 1,158, CT = 1,262, and TT = 414. Although a slight excess of homozygotes was observed (*P* = 0.021), genotype and allele frequencies of *RPTOR*-26934C/T were comparable to the previously reported values. No significant differences in male/female proportion and age distribution were observed among *UCP3*-55C/T genotype groups (*P* = 0.823, *χ*^2^ test). The *RPTOR*-26934C/T showed slight sex differences in genotype distribution (*P* = 0.024, *χ*^2^ test). Age distribution was not different between genotypes (*P* > 0.05, 1-way analysis of variance).

**Table 1:** **Characteristics of successfully genotyped individuals**

Traits	Male	Female
Numbers of individuals	1,533	1,301
Age	52.5 (9.3)	50.5 (8.6)
VFA (cm^2^)	114.2 (41.7)	62.5 (27.1)
WC (cm)	86.9 (8.5)	80.9 (9.3)
BMI (kg/m^2^)	24.3 (3.2)	22.7 (3.6)
FPG (mg/dl)	104.9 (20.1)	95.1 (13.2)
HbA_1c_ (%)	5.4 (0.6)	5.2 (0.5)
TG (mg/dl)	132.7 (88.3)	87.6 (47.6)
TC (mg/dl)	205.8 (32.0)	212.7 (34.8)
HDL-c (mg/dl)	57.7 (13.7)	70.6 (16.3)
LDL-c (mg/dl)	122.5 (28.3)	124.6 (30.7)

Except for the numbers of individuals, mean values (standard deviations) are shown.

BMI, body mass index; FPG, fasting plasma glucose, HbA_1c_, hemoglobin A_1c_; HDL-c, high density lipoprotein cholesterol; LDL-c, low density lipoprotein cholesterol; TC, total cholesterol; TG, triglycerides; VFA, visceral fat area; WA, waist circumference.

For both of *UCP3*-55C/T and *RPTOR-*26934C/T, the T allele was supposed to be linked with higher thermogenic activity since the allele frequency of the T allele was shown to be higher in ethnic groups at higher latitudes [14, 17]. Thus, the effects of T allele copy number (0, 1, or 2) on obesity-related quantitative traits were tested in each subject using multiple linear regression models (Table 2). We first tested the models comprising whole individuals. Mean values of BMI, WC, VFA and VFA adjusted for BMI were similar among genotype groups of both SNPs and no significant effect of the T allele copy number on the obesity-related traits was observed. Additionally, these two SNPs did not show associations with blood medical parameters including FPG, HbA_1c_, TG, TC, HDL-c and LDL-c (Table 3).

**Table 2:** **Association between obesity-related traits and the tested SNPs**

	CC		CT		TT		***P-***values
	Mean	SE	Mean	SE	Mean	SE	
*UCP3*-55C/T							
BMI (kg/m^2^)	23.60	0.09	23.50	0.10	23.52	0.20	>0.05
WC (cm)	84.24	0.24	84.08	0.26	84.13	0.54	>0.05
VFA (cm^2^)	90.60	0.90	90.48	0.95	90.01	2.00	>0.05
aVFA (cm^2^)	90.30	0.52	90.91	0.55	90.43	1.16	>0.05
*RPTOR*-26934C/T							
BMI (kg/m^2^)	23.47	0.10	23.60	0.10	23.60	0.16	>0.05
WC (cm)	84.21	0.26	84.16	0.25	84.06	0.43	> 0.05
VFA (cm^2^)	90.90	0.97	90.37	0.93	90.09	1.61	> 0.05
aVFA (cm^2^)	91.36	0.56	90.11	0.54	89.62	0.93	> 0.05

Mean values and the standard errors (SE) in each genotype group (CC, CT, and TT) are indicated. These values were adjusted for sex, age, age^2^, and walking speed. *P*-values of T allele copy numbers (0, 1, and 2) in multiple linear regression models are indicated.

aVFA, VFA adjusted for BMI; BMI, body mass index; VFA, visceral fat area; WA, waist circumference.

**Table 3:** **Associations between the tested SNPs and selected blood parameters**

Traits	***UCP3***-55C/T		***RPTOR***-26934C/T	
	Effects	***P***-values	Effects	***P***-values
FPG	−0.013	0.431	0.001	0.974
HbA_1c_	−0.022	0.210	−0.007	0.678
TG	0.005	0.779	−0.017	0.294
TC	0.030	0.105	0.015	0.410
HDL-c	−0.003	0.834	0.027	0.100
LDL-c	0.032	0.080	0.004	0.847

Effects (β coefficients) and *P*-values of T allele copy numbers (0, 1, and 2) in multiple linear regression models are indicated.

FPG, fasting plasma glucose, HbA_1c_, hemoglobin A_1c_; HDL-c, high density lipoprotein cholesterol; LDL-c, low density lipoprotein cholesterol; TC, total cholesterol; TG, triglycerides.

**Figure 1:**
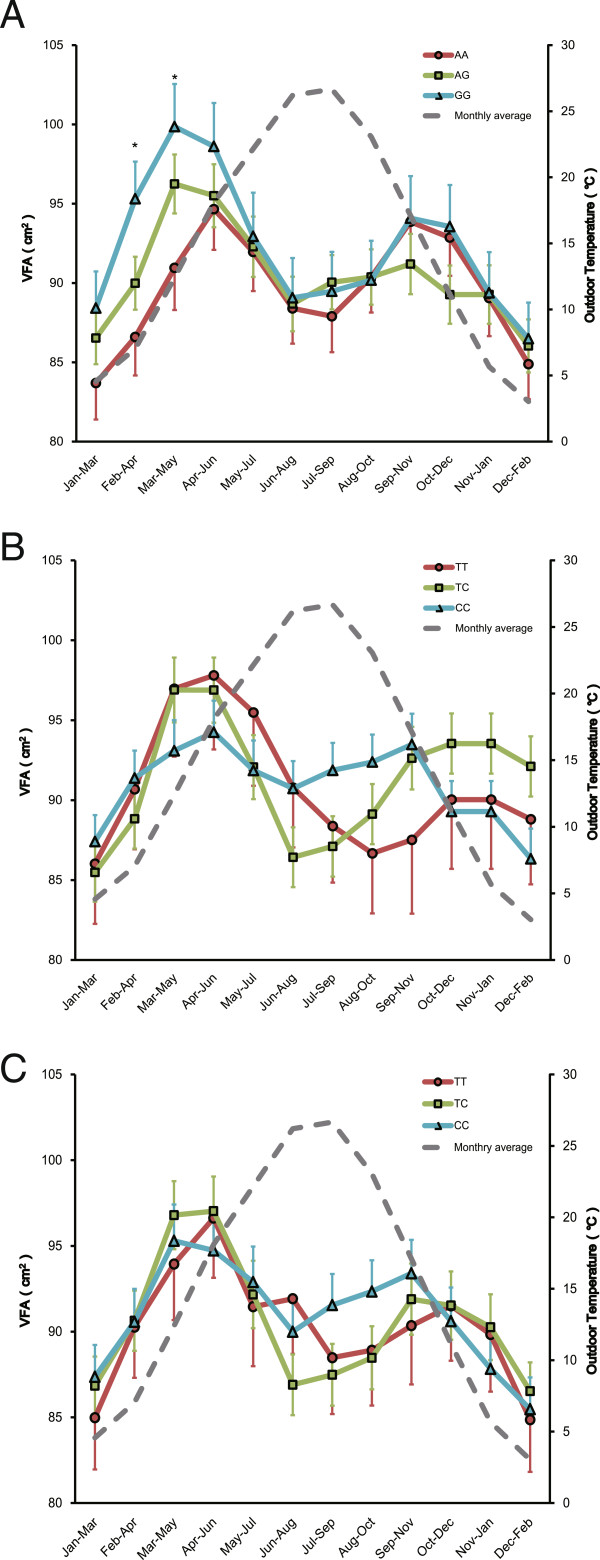
**Sliding window association analyses of the** ***UCP3-*** **-55C/T and the** ***RPTOR*** **-26934C/T.** Mean and standard errors of visceral fat area (VFA) by genotype groups in each 3-month subset are indicated. Broken lines indicate average outdoor mid-month temperature of each 3-month subset. **(A)** the data of *UCP1*-3826G/A was adopted from [11]; **(B)** *UCP3*-55C/T; **(C)** *RPTOR*-26934C/T. Asterisks indicate *P* < 0.05.

A previous study had revealed that the stratification of subjects by season of examination successfully refined the association between the *UCP1*-3826G/A and VFA [13]. This phenomenon was likely due to the physiological nature of BAT and VFA, wherein cold stress was required to enhance the NST, and VFA reflected the energy balance more sensitively than BMI or WC [11, 13]. If the *UCP3* and the *RPTOR* contributed to the efficiency of NST, a similar analysis would be useful for these 2 SNPs. Therefore, we assessed the seasonal effects of these two SNPs on the VFA using the sliding window sampling method. For the *UCP1*-3826G/A, carriers of the A allele showed smaller mean VFA during winter to spring, and the genotypic differences in VFA were significant in subsets sampled during February to May (Figure 1A, adopted from [13]). For *UCP3*-55C/T and *RPTOR*-26934C/T (Figure 1B and 1C), the mean values of VFA were very similar in these subsets (*P* > 0.10). Both SNPs showed a similar trend where the T allele showed smaller VFA during summer to autumn, but the associations were not significant (*P* > 0.098).

There are certain limitations to our study. Our large-scale cohort allowed us to test seasonality of the effect of SNPs; however, longitudinal analyses, instead of the sliding window analysis, may be more preferable to depict seasonal effect. Moreover, precise information about physical activity and dietary intakes, which should be controlled in the association analyses of obesity-related traits, was unavailable in our cohort. The present multiple linear regression models were not adjusted for skeletal muscle mass, which would have a large impact on obesity-related traits [22, 23]. Moreover, winter temperature of the study site (Kanto, Japan) was possibly not low enough to stimulate NST in skeletal muscle. Considering the nature of NST in skeletal muscle might be required to test the association of the *UCP3* and obesity-related traits. The present study tested a single SNP by a locus, but other SNPs influencing obesity-related traits have been reported for the *UCP3* and the *RPTOR* [24–27]. Therefore, the nearby SNPs and their haplotypes should be assessed for their association with obesity-related traits. Relevance of these two SNPs to NST must be tested by precise physiological genetic experiments using a climate chamber [28].

## Conclusions

Overall, our genetic association analyses including seasonality on a large-scale cohort showed that the *UCP3*-55C/T and the *RPTOR*-26934C/T did not influence obesity-related traits. Roles of the *UCP3* and the *RPTOR* in NST remain unclear but the effects of these two SNPs on energy metabolism balance and, perhaps, on efficiency of NST in humans may be subtle. Climate-associated natural selection on the *UCP3*-55C/T and the *RPTOR*-26934C/T can be explained by physiological functions other than NST. *UCP3* has been shown to be involved in the regulation of reactive oxygen species in mitochondria and, thus, may play a role in the maintenance of mitochondrial functions [29]. *RPTOR* encodes a subunit of the mammalian target of rapamycin (mTOR) complex 1 (mTORC1), which is a protein complex regulating protein synthesis in response to nutrition, energy, and cellular redox status [19]. The mTORC1 participates in various biological pathway including cell proliferation, angiogenesis, and various immune systems. Adaptation to pathogens, for instance, may explain the climate associated evolution of *RPTOR*-26934C/T [17]. Associations of these two SNPs with other various physiological traits should be tested in future. Finally, the signature of adaptive evolution for the *UCP3* and the *RPTOR* should be re-verified using molecular evolutionary methods other than those adopted by Hancock *et al*. and Sun *et al*. [14–17].

## Abbreviations

BAT: brown and beige/brite adipose tissues
BMI: body mass index
FPG: fasting plasma glucose
Hb: hemoglobin
HDL-c: high density lipoprotein cholesterol
LDL-c: low density lipoprotein cholesterol
mTORC1: mammalian target of rapamycin complex 1
NST: non-shivering thermogenesis
PCR: polymerase chain reaction
RPTOR: regulatory associated protein of mTOR complex 1
SNP: single nucleotide polymorphism
TC: total cholesterol
TG: triglycerides
UCP1: uncoupling protein 1
UCP3: uncoupling protein 3
VFA: visceral fat area
WC: waist circumference.
